# DDTRN: predicting bacterial transcriptional regulatory networks based on gene sequences using dual descriptor

**DOI:** 10.1093/bib/bbag534

**Published:** 2026-09-29

**Authors:** Pu Nie, Bin-Guang Ma

**Affiliations:** Hubei Key Laboratory of Agricultural Bioinformatics, College of Informatics, Huazhong Agricultural University, Wuhan 430070, China; Hubei Key Laboratory of Agricultural Bioinformatics, College of Informatics, Huazhong Agricultural University, Wuhan 430070, China

**Keywords:** transcriptional regulatory network, sequence-based prediction, dual descriptor model, bacterial gene regulation, position weight function

## Abstract

Accurate computational reconstruction of bacterial transcriptional regulatory network (TRN) remains a fundamental challenge in systems biology, particularly for non-model organisms lacking extensive transcriptomic data. We present DDTRN, a sequence-driven framework that formulates TRN inference as a binary classification task over concatenated regulator-target gene sequence pairs and employs a Dual Descriptor (DD) model to predict regulatory interactions. The DD architecture represents a sequence using two learnable components: Composition Weight Map (CWM) and Position Weight Function (PWF). We comprehensively evaluate DDTRN against six conventional machine learning (ML) baselines across eight benchmark bacterial datasets, including *Escherichia coli* (DREAM5, RegulonDB), *Bacillus subtilis*, *Salmonella enterica*, *Corynebacterium glutamicum*, *Mycobacterium tuberculosis*, *Pseudomonas aeruginosa*, and *Streptomyces coelicolor*. DDTRN achieves superior overall performance, attaining average area under the receiver operating characteristic curve and area under the precision–recall curve scores of 0.869 and 0.868, respectively, with particularly pronounced advantages at lower descriptor ranks where positional weighting compensates for limited sequence context. Systematic sensitivity analyses of rank, embedding dimension, and basis function count reveal stable optimal operating regimes, while subsampling experiments demonstrate strong robustness even with limited training data. Interpretability analyses show that PWF learns distinct periodic contributions across different rank granularities and that CWM preferentially weights meaningful *k*-mers. A case study on the *E. coli* dataset further illustrates that DDTRN identifies method-specific candidate targets complementary to those proposed by conventional approaches. By operating solely on genomic sequence, DDTRN provides a scalable, interpretable, and data-efficient framework for bacterial TRN inference in species where expression data are scarce, and it establishes a foundation for future multimodal integration with condition-specific regulatory information.

## Introduction

Transcriptional regulation is a fundamental process in cellular life, crucial for maintaining normal cell growth and function [1]. This process modulates transcriptional efficiency by regulating the activity of RNA polymerase or its associated transcription factor (TF) proteins, thereby influencing gene expression levels and involving virtually every basic activity of life [2]. The transcriptional regulatory network (TRN), which delineates the regulatory relationships between TFs and target genes (TGs), is often regarded as a functionally upstream regulatory core because it directly controls the initiation and intensity of gene expression [3–5]—a complex, multi-step process that converts specific DNA sequences into functional proteins through precisely regulated stages including transcription, RNA processing, and translation [6]. In the early stages of genomics, reconstructing genome-scale TRNs was a highly challenging systems engineering task, with the core difficulty lying in the heavy reliance on massive experimental data to identify transcription factor binding sites (TFBS) and characterize their regulatory activity [7]. However, with the advent and rapid development of high-throughput sequencing technologies in the post-genomic era [8], it has become feasible to readily acquire genomic sequence data and gene expression profiles. Concurrently, continuous advancements in machine learning (ML) have opened new technical pathways for constructing high-precision TRNs [9].

Early TRN construction primarily relied on biological experiments such as ChIP-seq to validate regulatory relationships between TFs and TGs on a case-by-case basis. These techniques enabled genome-scale mapping of TFBS, laying a crucial foundation for building large-scale regulatory datasets and subsequent computational modeling [10]. Furthermore, *in vitro* high-throughput assays such as DAP-seq, owing to their speed, low cost, and scalability, have provided important means for large-scale TFBS acquisition [11]. When large-scale gene expression data are available, predicting TRNs using RNA-seq data as the core input has become a mainstream approach, leading to the development of a series of computational methods. For instance, GRADIS employs a support vector machine (SVM) to reconstruct TRNs based on graph representations of transcriptomic data [12]; GRGNN formulates TRN prediction as a graph classification problem, utilizing graph neural networks for end-to-end learning [13]; the recently proposed PGBTR is a general deep learning framework for bacterial TRN prediction that integrates expression data and genomic information [14]. Based on the complex-valued modeling approaches [15–18], RNDEtree combines nonlinear ordinary differential equations with a flexible neural tree for gene regulatory network inference [19]. However, expression-based TRN inference methods typically depend on large-scale transcriptomic data, which can be affected by noise, batch effects, and heterogeneity. More critically, for many non-model organisms, sufficient condition-specific RNA-seq data are often unavailable, limiting the applicability of transcriptome-dependent TRN inference methods in data-scarce scenarios [12–14, 19].

Another core aspect of regulatory prediction is the modeling and characterization of TFBS and their sequence motifs. Traditional methods often use position weight matrices to represent sequence specificity, but their expressive power is limited. Deep learning enables end-to-end learning from raw DNA sequences, significantly enhancing predictive capabilities: DeepBind uses Convolutional Neural Networks (CNNs) to learn the sequence-binding specificity of proteins [20]; DeepSEA takes sequences as input to simultaneously predict multiple regulatory phenotypes, demonstrating the feasibility of sequence-based prediction [21]; DanQ combines CNNs and Bidirectional Long Short-Term Memory networks to model motif features and long-range dependencies [22]; Enformer further integrates long-range genomic interaction information to improve prediction performance [23]. These advances indicate that sequences contain rich regulatory information, making it possible to predict regulatory relationships using sequence as the primary input even in the absence of expression data [20–23]. Therefore, how to construct a model capable of stably predicting genuine regulatory relationships relying solely on widely available genomic sequence information remains a worthwhile research challenge.

Building upon these challenges and opportunities, this work proposes a novel TRN prediction method based on the Dual Descriptor (DD) model [24], termed DDTRN. This method predicts regulatory relationships between TFs and TGs using only gene sequence information, thereby providing a sequence-driven alternative to methods that depend on transcriptomic or other omics data. We evaluated DDTRN against other machine learning methods across eight benchmark datasets. Overall, DDTRN achieved the best performance on most datasets. Moreover, compared to traditional ML models, DDTRN provides an interpretable representation of sequence information: the model decomposes sequence information into compositional and arrangement information, characterized by the Composition Weight Map (CWM) and Position Weight Function (PWF), respectively, thereby providing an interpretable view of how compositional and positional sequence patterns relate to transcriptional regulation.

## Materials and methods

### Benchmark datasets

DDTRN was evaluated on eight bacterial TRN datasets, including the *Escherichia coli* dataset from the DREAM5 challenge [25], two datasets constructed by Gu and Ma [14] (RegulonDB_Ecoli, Subtiwiki_Bsubtilis), one dataset from iModulonDB 2.0 [26] (*Salmonella enterica*), and four datasets from Abasy Atlas [27] (*Corynebacterium glutamicum*, *Mycobacterium tuberculosis*, *Pseudomonas aeruginosa*, *Streptomyces coelicolor*). The gene sequences for these bacterial species corresponding to the eight TRN datasets were extracted from reference genomes obtained from NCBI Datasets [28]. Table 1 displays detailed information for the datasets used in this study.

**Table 1:** Information on the datasets used in this study.

**Dataset**	**Gene no.**	**Regulation no.**	**TF no.**
Dream5_Ecoli	4297	2066	334
RegulonDB_Ecoli	4270	5316	220
Subtiwiki_Bsubtilis	4325	3033	185
*S. enterica*	2068	1808	75
*C. glutamicum*	2228	3013	71
*M. tuberculosis*	3056	9813	179
*P. aeruginosa*	2627	4390	91
*S. coelicolor*	4863	5024	160

### D‌DTRN model architecture based on dual descriptor

DDTRN was designed as a sequence-driven framework for predicting bacterial transcriptional regulatory relationships from genomic sequences (Fig. 1). Based on the benchmark datasets, the DNA sequences of the genes with regulatory relationships in the bacterial TRNs were prepared as the input data. For each candidate TF–TG pair, the DNA sequence of the TF gene and that of the TG were concatenated into a single composite sequence (Fig. 1A), which was then represented by the vector DD model (tensor form), termed DualDescriptorTS [29]. The final task was formulated as a binary classification problem, where each composite sequence was assigned to either the regulatory or non-regulatory class.

**Figure 1:**
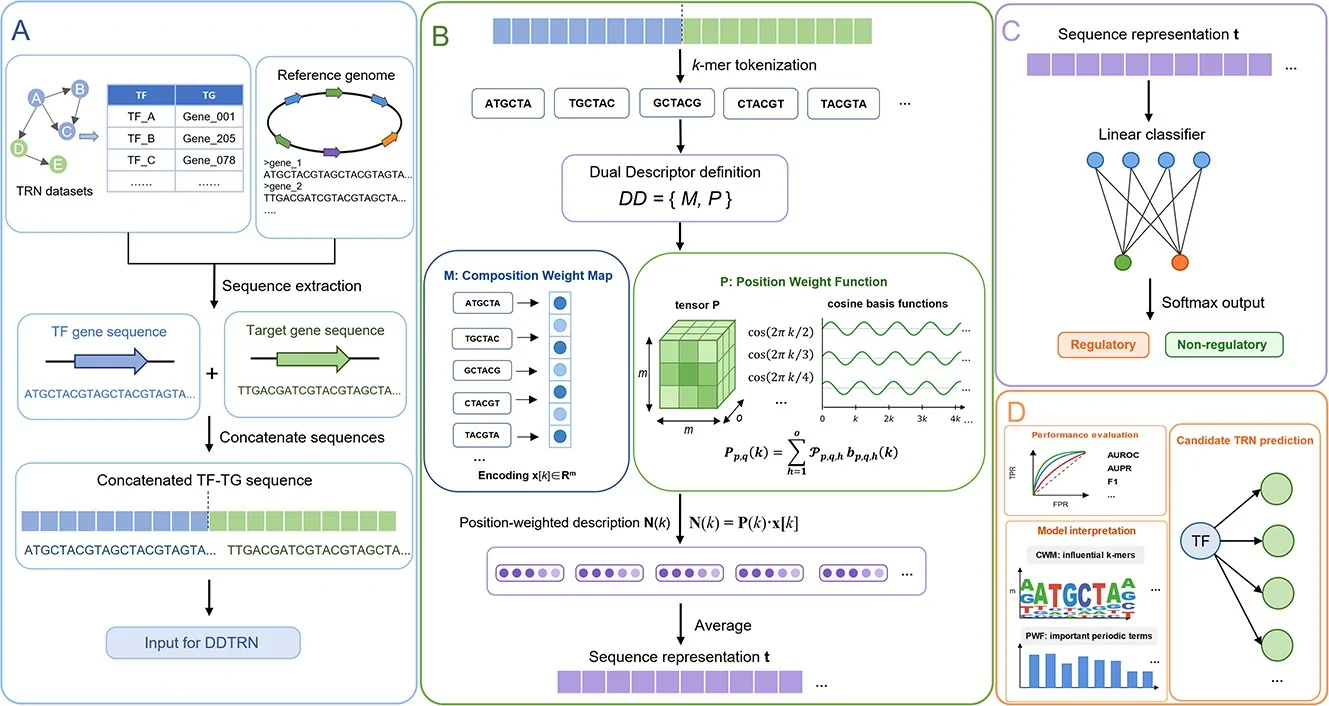
The workflow of DDTRN; (A) construction of concatenated gene sequences from TF and TG genes; (B) definition of DD for sequence representation; (C) regulatory relationship prediction; (D) model evaluation and interpretation.

The core of DDTRN is the DD representation: *DD* = {*M*, *P*}, which decomposes sequence information into two complementary components: the CWM (namely *M*) and the PWF (namely *P*) [30]. The CWM describes the compositional contribution of sequence tokens, while the PWF describes their positional or arrangement-dependent contribution (Fig. 1B). In this way, DDTRN differs from conventional *k*-mer frequency-based representations, which mainly encode how often each *k*-mer occurs but do not explicitly model where and how these sequence fragments are arranged.

Given an input sequence, DD first tokenizes it into rank-length nucleotide fragments. When the descriptor rank is *r*, each token corresponds to an *r*-mer over the alphabet {A, C, G, T}. In the default linear mode, tokens are extracted using a sliding window with stride 1, allowing overlapping *r*-mers to preserve fine-grained local sequence patterns. Each valid *r*-mer token is then mapped by the CWM to a learnable embedding vector $\mathbf{x}\left[k\right]\in{R}^m$, where *m* is the encoding vector dimension and *k* denotes the token position in the sequence.

To incorporate positional information, DD uses a learnable position-weight tensor $\mathbf{P}\in{R}^{m\times m\times o}$, where *o* is the number of basis functions used to expand the PWF. For each token position *k*, a set of cosine basis functions is computed as:


(1) \begin{equation*} {b}_{p,q,h}(k)=\cos \left(\frac{2\pi k}{\mathrm{period}\left[p,q,h\right]}\right) \end{equation*}

where period[*p*, *q*, *h*] is the indexed period, with *p*, *q*, *h* indicating the indices in the three dimensions of tensor **P**. In the implementation, the period is defined as:


(2) \begin{equation*} {\displaystyle \begin{array}{c}\mathrm{period}\left[p,q,h\right]=p\times \left(m\times o\right)+q\times o+h+2\end{array}} \end{equation*}

The position-weighted description vector **N**(*k*) for the *k*-th token is then calculated by combining the token embedding, the position weight tensor, and the corresponding cosine basis functions:


(3) \begin{equation*} {\displaystyle \begin{array}{c}{N}_p(k)=\sum_{q=1}^m\sum_{h=1}^o{P}_{p,q,h}\cdot{b}_{p,q,h}(k)\cdot{x}_q^p\left[k\right]\kern2em \left(p=1,2,\dots, m\right)\end{array}} \end{equation*}

Thus, each **N**(*k*) vector jointly reflects the identity of the local sequence fragment and its position-dependent contribution within the composite TF–TG sequence. The representation of the whole sequence is obtained by averaging all position-level description vectors:


(4) \begin{equation*} {\displaystyle \begin{array}{c}\mathbf{t}=\frac{1}{L}\sum_{k=1}^L\mathbf{N}(k)\end{array}} \end{equation*}

where *L* is the number of extracted tokens. This average description vector **t** serves as the latent representation of the candidate TF–TG pair.

For regulatory relationship prediction, a linear classification head was attached to the DD representation (Fig. 1C). The classifier maps **t** to two output logits corresponding to the regulatory and non-regulatory classes, and the probability of a regulatory interaction is obtained using the softmax function. During training, the parameters of the CWM, the PWF tensor, and the classification head were optimized jointly by minimizing the cross-entropy loss. Therefore, DDTRN learns sequence representations in an end-to-end manner while retaining the interpretability of the DD framework: the CWM parameters indicate which *r*-mer tokens contribute strongly to the prediction, whereas the PWF tensor reflects which positional or periodic components are important for encoding regulatory sequence patterns.

In this study, the default DD configuration was set to *rank* = 6, *rank_mode* = “drop”, encoding vector dimension *m* = 10, number of basis functions *o* = 10, and description *mode* = linear/nonlinear. These values were selected based on the parameter sensitivity analyses described below. The model was implemented in PyTorch and trained on a GPU when available [29].

### Baseline methods

For the baseline methods, six classifiers were employed with their standard implementations: SVM [31] with a radial basis function kernel via SVC(probability = True), Random Forest (RF) [32] with max_features = “sqrt”, Gradient Boosting Decision Trees (GBDT) [33] with early stopping (n_iter_no_change = 10, validation_fraction = 0.1), Light Gradient Boosting Machine (LGBM) [34] with learning_rate = 0.5, eXtreme Gradient Boosting‌ (XGBoost) [35] with early_stopping_rounds = 10, Multilayer Perceptron (MLP) [36] with one hidden layer of 100 units, max_iter = 500, and early stopping. The six baseline classifiers were selected to represent complementary supervised-learning paradigms while enabling comparison using the same *k*-mer frequency representation. The same concatenated TF–TG gene sequences were transformed into fixed-dimensional feature vectors based on *k*-mer frequency counts. Specifically, all possible *k*-mers of length rank were enumerated from the alphabet {A, C, G, T} to form a vocabulary of size 4^rank^. For each sequence, a sliding window of length rank (stride = 1, i.e. linear mode) was used to extract all overlapping *k*-mers; only those fully composed of A, C, G, or T were retained. The occurrence count of each *k*-mer was then normalized by the total number of valid *k*-mers in the sequence, producing a frequency vector of dimension 4^rank^. For SVM and MLP, standardscaler was applied as a preprocessing step within a pipeline to normalize the *k*-mer frequency features. All models used the same random seed for reproducibility.

### Training and testing strategies

A balanced dataset was constructed for each bacterial species by pairing all known positive regulatory interactions (TF–TG) with an equal number of negative samples. Negative samples were generated using a TF-based strategy: for each TF, a TG was randomly selected from the set of all genes that are neither the TF itself nor a known target of that TF, ensuring that the negative pair did not appear in the positive set. It is worth noting that, because current bacterial TRN annotations remain incomplete, some unannotated TF–TG pairs treated as putative negative samples may actually represent regulatory interactions that have not yet been discovered. The entire set of labeled pairs (positive and negative) was randomly shuffled and then split into training (80%) and testing (20%) sets using stratified sampling to preserve the class distribution. From the training set, an additional 20% was held out as a validation set, again via stratified random sampling. For DDTRN, the validation set was used for early stopping and hyperparameter tuning. The training procedure employed the Adam optimizer with an initial learning rate of 1.5 × 10^−4^, a batch size of 32, and an exponential learning rate scheduler with a decay factor (*γ*) of 0.99 per epoch. The model was trained for up to 300 epochs with early stopping patience of 10 epochs based on validation loss; the model parameters yielding the lowest validation loss were restored. For the baseline methods, the same training/validation/test splits were used. Classifiers supporting early stopping (XGBoost, LGBM, GBDT, and MLP) were provided with the validation set to terminate training when performance ceased to improve. All other classifiers were trained on the training set without validation intervention. The random seed was fixed for all experiments to ensure reproducibility. To assess the robustness of the evaluation, all methods were additionally repeated 20 times using random seeds 0–19, and the results are presented in Supplementary Table S1.

### Metrics for evaluation

The performance of DDTRN and all baseline methods was assessed using six standard classification metrics computed on the held-out test set (Fig. 1D). Accuracy was defined as the proportion of correctly classified instances. Precision (positive predictive value) measured the fraction of predicted positive interactions that were true positives, while recall (sensitivity) measured the fraction of actual positive interactions that were correctly identified. The F1 score, the harmonic mean of precision and recall, provided a balanced measure. In addition, two threshold-independent metrics were used: the area under the receiver operating characteristic curve (AUROC) and the area under the precision–recall curve (AUPR). For AUROC and AUPR, the predicted probability of the positive class (regulatory interaction) was used as the decision score. In DDTRN, these probabilities were obtained via the softmax output of the classifier head; in baseline methods, they were obtained either from predict_proba (for classifiers that support it) or from decision_function (for SVM). All metrics were computed using the scikit-learn library.

### Interpretability analysis

To interpret the learned features underpinning DDTRN’s predictive performance, we systematically analyzed the two core components of the DD: the CWM (parameter tensor **M**) and the PWF (parameter tensor **P**). Both sets of parameters are learned end-to-end during training without additional supervision, and their structures provide direct mechanistic insight into how sequence composition and positional arrangement contribute to regulatory prediction (Fig. 1D).

For CWM analysis, we focused on the encoding vectors assigned to each possible *r*-mer token (e.g. *rank* = 6 in default settings). For each token, we computed the L2 norm of its corresponding embedding vector as a proxy for its overall influence on the sequence representation. Tokens were then ranked by this norm, and the top-ranking *r*-mers were examined for sequence-level patterns. This analysis aimed to identify which short sequence fragments the model consistently treated as informative for distinguishing regulatory from non-regulatory TF–TG pairs.

For PWF analysis, we examined the learned tensor **P** of shape *m* × *m* × *o*, where each element *P_pqh_*​ modulates the contribution of a specific cosine basis function with period defined in Equation (2). To quantify the relative importance of each periodic component, we calculated the absolute value |*P_pqh_*| and ranked the corresponding basis terms accordingly. This procedure was performed separately for different rank settings (1–6) under both linear and nonlinear token extraction modes. Larger |*P_pqh_|* values indicate stronger reliance on particular periodic positional patterns during sequence encoding. By comparing the distribution and magnitude of these values across ranks, we assessed how the model compensates for limited local sequence information with positional weighting.

The interpretability analysis was conducted on the trained DDTRN models using the Dream5_Ecoli dataset. All visualizations, including bar plots for top-ranked *r*-mers and heatmaps for periodic terms, were generated using Matplotlib.

## Results

### Performance evaluation

To evaluate the performance of DDTRN in inferring bacterial TRNs, we conducted a comparative analysis against six baseline methods (SVM, RF, GBDT, LGBM, XGBoost, and MLP) across the eight datasets. For these machine learning methods, we used *k*-mer frequency vectors as the feature representation for the concatenated sequences. A sliding window with a stride of 1 was used to extract all *k*-mers, count the occurrences of each *k*-mer, and normalize them to frequencies, resulting in a feature vector of size 4^rank^ (where the *k*-mer length corresponds to the “rank” parameter in DDTRN). All methods were evaluated using a consistent evaluation strategy and the same test sets.

As shown in Table 2, the predictive performance of DDTRN and six baseline machine learning methods at *rank* = 6 was evaluated across eight benchmark datasets. Overall, DDTRN achieved the best average performance among all methods, with an average AUROC of 0.8690, an average AUPR of 0.8683, and an average F1 score of 0.8053. These results indicate that DDTRN provides the most balanced overall performance across different bacterial TRN datasets. Furthermore, on the *M. tuberculosis* dataset, which contains the largest number of known regulatory interactions among the benchmark datasets, DDTRN consistently outperformed all baseline methods across all metrics. This suggests that DDTRN remains effective in more complex and densely connected regulatory networks. While some baseline methods demonstrate competitiveness on specific datasets, such as SVM on the RegulonDB_Ecoli dataset, and GBDT or LGBM on some other datasets, their strengths are often limited to specific datasets or metrics. In contrast, DDTRN exhibits more stable overall performance across a wide range of datasets, ranking highly in average AUROC, AUPR, and F1 scores. This suggests that the sequence representations learned by DDTRN are more consistently able to capture regulatory information than traditional *k*-mer frequency-based machine learning models. Additionally, all methods were repeated 20 times using random seeds 0–19, showing generally stable performance across runs (Supplementary Table S1).

**Table 2:** Performance of various methods on the eight benchmark datasets.

**Dataset**	**Metric**	**DDTRN**	**SVM**	**GBDT**	**LGBM**	**RF**	**XGBoost**	**MLP**
Dream5_Ecoli	AUROC	**0.8213**	0.8005	0.7886	0.7861	0.7869	0.7814	0.7620
	AUPR	**0.8296**	0.7934	0.8085	0.8031	0.8097	0.7997	0.7686
	F1	**0.7429**	0.7277	0.7141	0.7031	0.7039	0.7177	0.6916
RegulonDB_Ecoli	AUROC	0.8441	**0.8681**	0.8494	0.8420	0.8533	0.8486	0.8388
	AUPR	0.8523	**0.8726**	0.8598	0.8470	0.8626	0.8569	0.8353
	F1	0.7699	**0.7892**	0.7692	0.7701	0.7579	0.7756	0.7643
Subtiwiki_Bsubtilis	AUROC	0.8299	**0.8357**	0.8159	0.8169	0.8177	0.8024	0.7868
	AUPR	0.8309	**0.8465**	0.8259	0.8180	0.8230	0.8063	0.8004
	F1	**0.7578**	0.7454	0.7298	0.7506	0.7201	0.7325	0.7138
*S. enterica*	AUROC	**0.8703**	0.8450	0.8568	0.8435	0.8597	0.8453	0.8060
	AUPR	**0.8881**	0.8702	0.8800	0.8657	0.8831	0.8729	0.8314
	F1	**0.8063**	0.7953	0.7674	0.7734	0.7680	0.7616	0.7535
*C. glutamicum*	AUROC	0.9218	0.9224	0.9216	**0.9245**	0.9211	0.9128	0.9035
	AUPR	**0.9366**	0.9258	0.9304	0.9334	0.9329	0.9289	0.9169
	F1	0.8490	**0.8546**	0.8472	0.8464	0.8449	0.8402	0.8305
*M. tuberculosis*	AUROC	**0.8263**	0.8227	0.8145	0.7873	0.8054	0.8005	0.8090
	AUPR	**0.7918**	0.7887	0.7895	0.7646	0.7716	0.7766	0.7818
	F1	**0.7679**	0.7665	0.7420	0.7185	0.7363	0.7335	0.7365
*P. aeruginosa*	AUROC	0.8885	0.8823	**0.8906**	0.8861	0.8827	0.8800	0.8617
	AUPR	0.8756	0.8645	**0.8886**	0.8744	0.8757	0.8624	0.8305
	F1	**0.8327**	0.8243	0.8211	0.8229	0.8167	0.8134	0.8078
	AUROC	0.9497	0.9372	0.9520	**0.9521**	0.9502	0.9508	0.9410
*S. coelicolor*	AUPR	0.9417	0.9209	**0.9495**	0.9407	0.9343	0.9437	0.9218
	F1	0.9162	0.8979	0.9127	**0.9178**	0.9157	0.9120	0.8937
	AUROC	**0.8690**	0.8642	0.8612	0.8548	0.8596	0.8527	0.8386
**Average**	AUPR	**0.8683**	0.8603	0.8665	0.8559	0.8616	0.8559	0.8358
	F1	**0.8053**	0.8001	0.7879	0.7878	0.7829	0.7858	0.7740

Furthermore, the results in Supplementary Fig. S3 show that DDTRN has a more significant advantage at lower ranks (*rank* = 1, 2, 3). This trend can be explained by differences in sequence representation strategies. Traditional machine learning methods use *k*-mer frequency vectors to represent concatenated TF–TG sequences; these vectors primarily encode compositional information while largely ignoring positional arrangement. Therefore, these methods have limited ability to capture long-range sequence structure at lower ranks. In contrast, the DD representation used in DDTRN integrates CWM and PWF components, enabling the model to simultaneously encode nucleotide composition and positional information. The learnable PWF components assign different weights to different sequence positions, thus compensating for the limited structural information contained in short *k*-mers. As the rank increases, the *k*-mer fragments themselves contain more permutation information, allowing traditional *k*-mer frequency-based methods to gradually improve. Therefore, the performance gap between DDTRN and baseline methods gradually narrows at higher ranks. The subsequent PWF parameter analysis also supports this explanation: at low ranks, the |*P_pqh_*| values ​​of the first few terms are significantly larger, indicating a greater reliance on positional weights to enhance sequence encoding; as the rank increases, the |*P_pqh_*| values ​​generally stabilize, and the contribution of PWF gradually weakens.

### Parameter analysis

The performance of the DDTRN model is influenced by several parameters, including descriptor rank (“rank”), vector embedding dimension (“vec_dim”), and the number of basis functions used for expanding the PWF (“num_basis”). To assess the model’s effectiveness in predicting TRNs, we compared the trends of AUROC, AUPR, and F1 across the eight benchmark datasets, varying one parameter while keeping the other two constant to adhere to the principle of univariate analysis. We systematically varied each parameter to evaluate its effect on predictive performance on TRN prediction.

#### Sensitivity analysis of rank

In DDTRN, rank determines the length of the sequence fragments (tokens) extracted from tandem TF–TG gene sequences. When *rank* = *k*, the model uses *k*-mer level tokens to represent sequences and applies weights to these tokens based on their position before aggregating them into a description vector. Therefore, the value of rank directly affects the model’s ability to capture local sequence patterns related to regulatory relationships.

To investigate the impact of this parameter, we evaluated DDTRN with rank values ​​of 1, 3, 6, 9, and 11 on eight benchmark datasets. As shown in Fig. 2, performance generally improves significantly as rank increases from 1 to 6. This trend was observed across all datasets for the AUROC, AUPR, and F1 metrics, indicating that short tokens with low rank values ​​provide insufficient local sequence information, while *k*-mers of moderate length are more effective in capturing regulatory sequence patterns in tandem TF–TG gene sequences. When rank is further increased from 6 to 9 or 11, the performance improvement decreases significantly and tends to stabilize. This suggests that beyond a certain limit, increasing token length does not consistently introduce proportionally effective information. Instead, longer *k*-mers may increase feature sparsity, generating rarer or noisy token combinations and increasing computational complexity.

**Figure 2:**
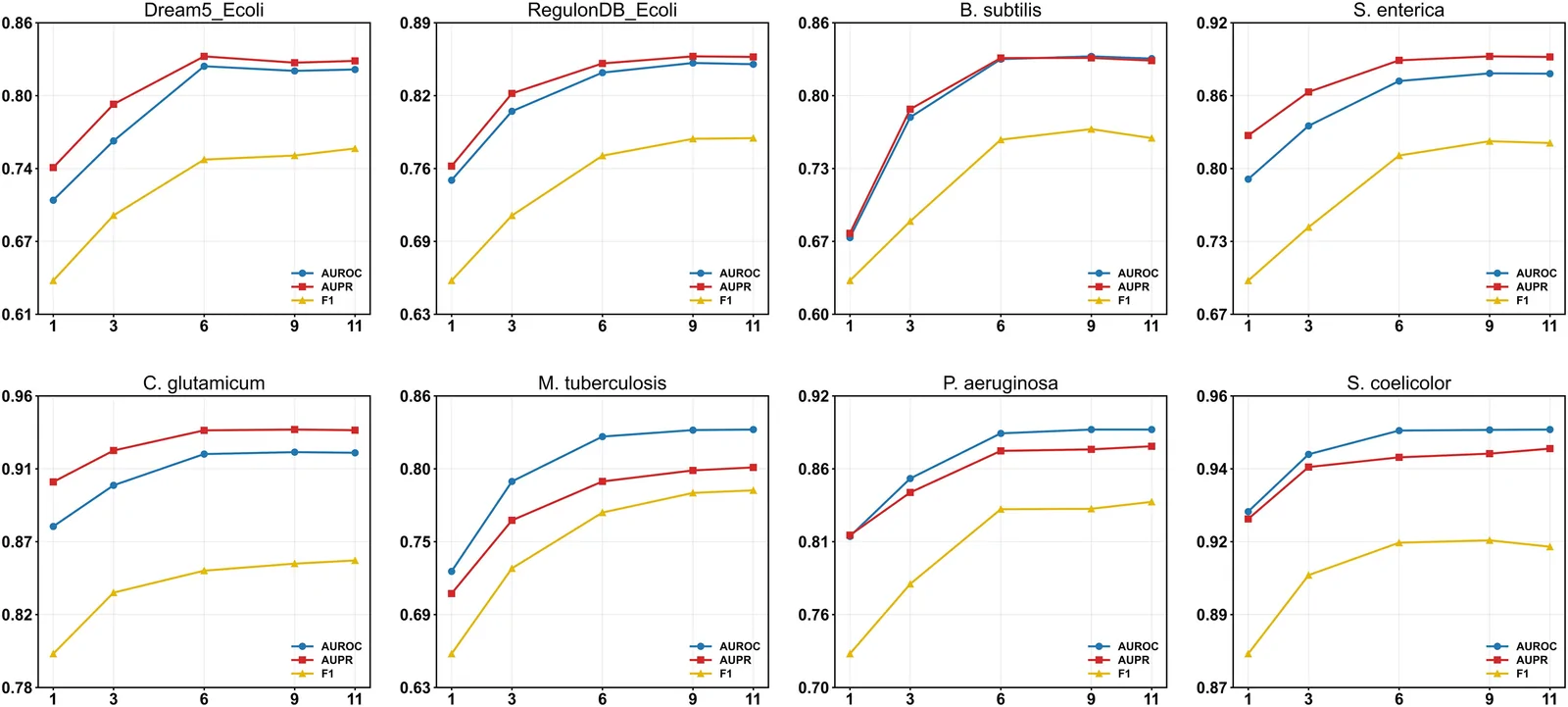
Sensitivity analysis for parameter “rank”; performance generally increases with rank and *rank* = 6 is a good choice and adopted to show the prediction performance.

Considering both prediction performance and computational efficiency, we chose *rank* = 6 as the default setting for DDTRN. This value captures sufficient local sequence information while avoiding increased computational complexity due to excessively high rank values. Therefore, *rank* = 6 represents the optimal choice for model accuracy, stability, and computational cost in subsequent experiments.

#### Sensitivity analysis of vec_dim

The vec_dim parameter determines the dimensionality of the encoding vector representation in DDTRN and affects the expressive capacity of the sequence descriptor. We evaluated vec_dim values of 2, 4, 6, 8, 10, and 12 across eight datasets. As shown in Supplementary Fig. S1, performance generally improved when vec_dim increased from 2 to 4 or 6, indicating that very low-dimensional embeddings are insufficient to capture regulatory sequence patterns. After vec_dim reached 6–8, AUROC, AUPR, and F1 tended to stabilize in most datasets, and further increasing the dimension to 10 or 12 brought only slight improvement. Therefore, we selected *vec_dim* = 10 as the default setting, as it provides stable near-optimal performance while maintaining sufficient representational capacity and acceptable computational complexity.

#### Sensitivity analysis of num_basis

The num_basis parameter controls the number of basis functions used to construct the PWF, thus affecting the granularity of positional encoding in DDTRN. We evaluated num_basis values ​​of 2, 4, 6, 8, 10, and 12 on eight datasets. As shown in Supplementary Fig. S2, the AUROC, AUPR, and F1 scores change little with the num_basis value, indicating that DDTRN is relatively insensitive to this parameter and maintains stable performance over a wide range of basis function numbers. While no sustained monotonic improvement was observed, the F1 score improved slightly on some datasets when num_basis was increased to around 10, such as Dream5_Ecoli, Subtiwiki_Bsubtilis, *S. enterica*, and *P. aeruginosa*. Further increasing num_basis to 12 did not yield significant additional improvements and even showed slight fluctuations on some datasets. Therefore, we chose *num_basis* = 10 as the default setting for this study.

#### Robustness of DDTRN

To evaluate the impact of training data volume on model performance, we randomly sampled 10%, 20%, 30%, 40%, 50%, and 60% of the training data from eight datasets, while maintaining the independence of the validation and test sets. Each setting was repeated using a different random seed, and box plots were used to summarize AUROC and AUPR values. As shown in Fig. 3, DDTRN maintained stable performance on most datasets as the training data size varied. On some datasets, even at lower training ratios, the model achieved relatively high AUROC and AUPR values, and the results of repeated runs were relatively concentrated. For Dream5_Ecoli, Subtiwiki_Bsubtilis, and *S. enterica* datasets, performance significantly improved when the training ratio increased from 10% to 20% or 30%, then gradually stabilized. These results demonstrate good robustness and performance even on a small number of sampled training datasets.

**Figure 3:**
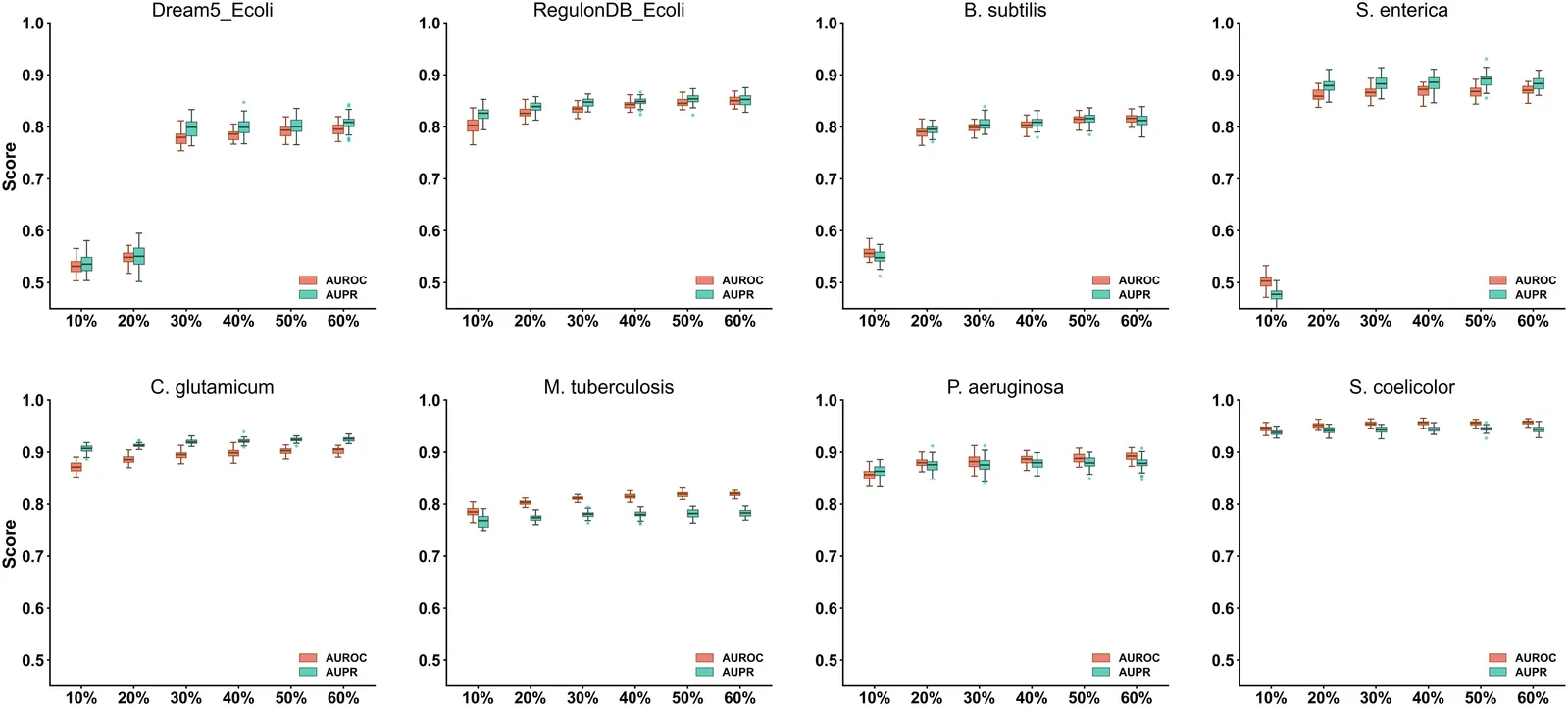
Robustness analysis for data size; generally, DDTRN is not sensitive to data size variation, and on most datasets, 20%–30% sample size is enough for good prediction.

#### Parameter-freezing experiments

Since CWM and PWF jointly define the DD representation rather than operating as independent, detachable modules, directly removing either component would alter the basic formulation of DD. Therefore, a conventional ablation study is not suitable in this case. Alternatively, we evaluated the contributions of CWM and PWF through parameter-freezing experiments while preserving the complete DD formulation. In the fixed-CWM setting, the CWM was randomly initialized and kept fixed while the PWF was trained; conversely, in the fixed-PWF setting, the PWF was randomly initialized and kept fixed while the CWM was trained. As shown in Supplementary Table S2, jointly optimizing CWM and PWF generally yielded better AUROC and AUPR than fixing either component. Notably, at ranks 1–5, the fixed-CWM setting consistently outperformed the fixed-PWF setting in AUROC and AUPR, indicating that learning the PWF is particularly important when short *k*-mers provide limited sequence information. In contrast, at *rank* = 6, the fixed-PWF setting outperformed the fixed-CWM setting, suggesting a relatively greater contribution of learnable compositional information when longer *k*-mers contain richer sequence information.

### Interpretability analysis

#### Interpretation of composition weight map

In DDTRN, M corresponds to the learnable parameters of the CWM, which assigns a vector representation to each token of length rank. When *rank* = 6, there are 4^6^ = 4096 possible 6-mers, and each 6-mer is associated with a learnable embedding vector. To estimate the relative contribution of different 6-mers, we calculated the L2 norm of each embedding vector and ranked all 6-mers accordingly. As shown in Fig. 4, the top 20 6-mers with the largest M values were identified under the nonlinear mode at *rank* = 6. The CWM parameter M mainly captures compositional patterns that contribute strongly to sequence representation. The top-ranked 6-mers, such as GCGACG, GTAGTT, AGTATG, and CGCGTG, exhibited relatively large embedding norms, indicating that these fragments had a stronger influence on the learned sequence representation.

**Figure 4:**
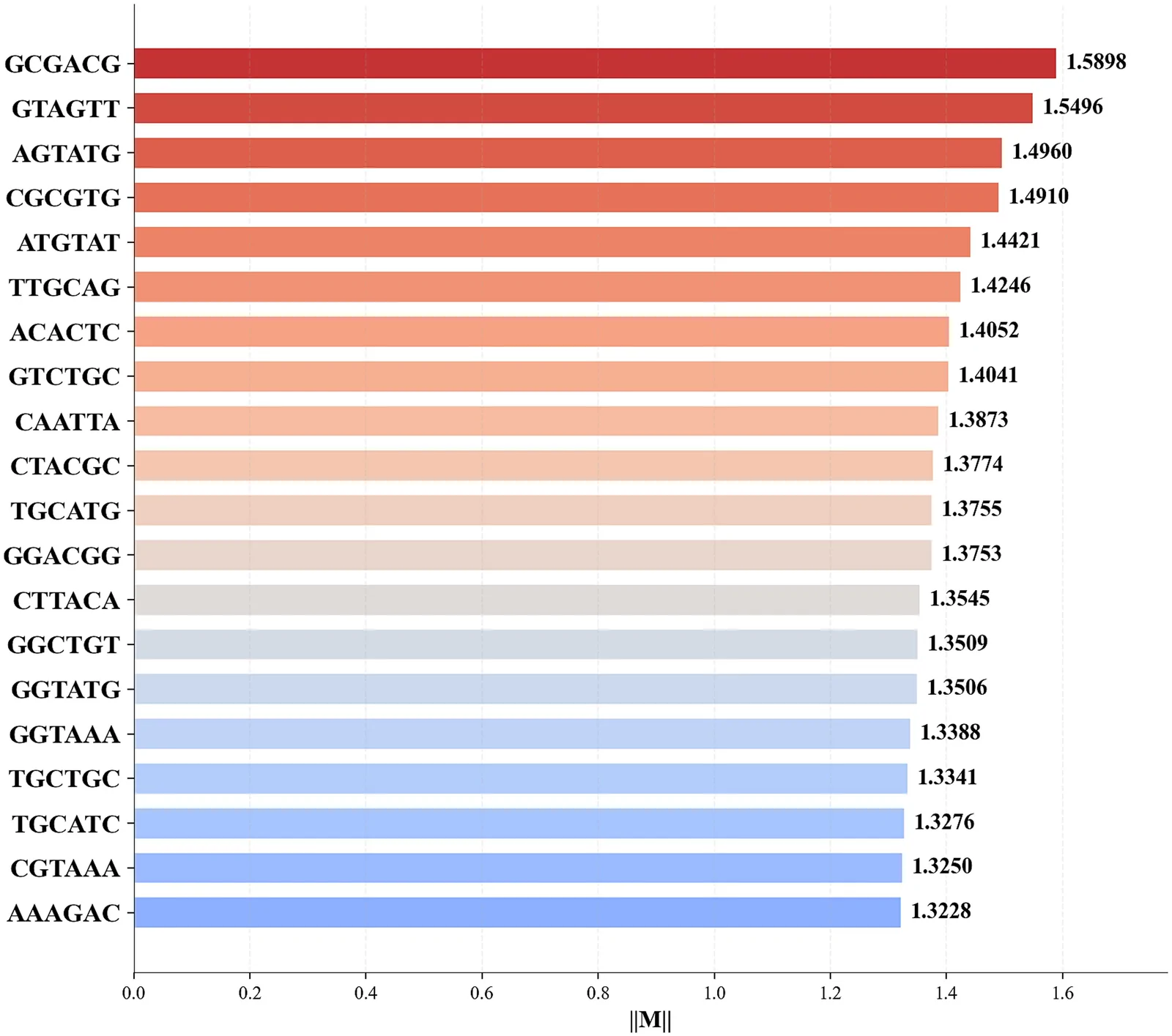
The top 20 most influential 6-mer tokens learned by CWM; the bar chart represents the length (norm) of each 6-mer embedding vector.

To characterize these high-norm 6-mers, we translated the top-ranked 6-mer tokens into amino-acid residues and examined whether specific residue categories were enriched. As shown in Fig. 5, using the *E. coli* genomic amino-acid background calculated from coding sequences, the combined frequency of Cys and Met was 3.97% (53 680/1 353 650). In contrast, the translated residues of the top 20 6-mers contained 10 Cys/Met residues out of 40 total residues, whereas only 1.59 residues would be expected based on the *E. coli* genome background. This corresponds to a 6.3-fold enrichment, with a significant enrichment signal after multiple-testing correction (one-sided binomial test‌ *P* = 3.00 × 10^−6^, *FDR* = 1.40 × 10^−5^). Similar significant enrichment was also observed in larger top-ranked sets (e.g. top 50, top 100), suggesting that sulfur-containing amino-acid-associated codon patterns are preferentially represented among the highest-weighted CWM tokens. The biophysical/biochemical mechanisms underlying this enrichment require further investigation to be clarified.

**Figure 5:**
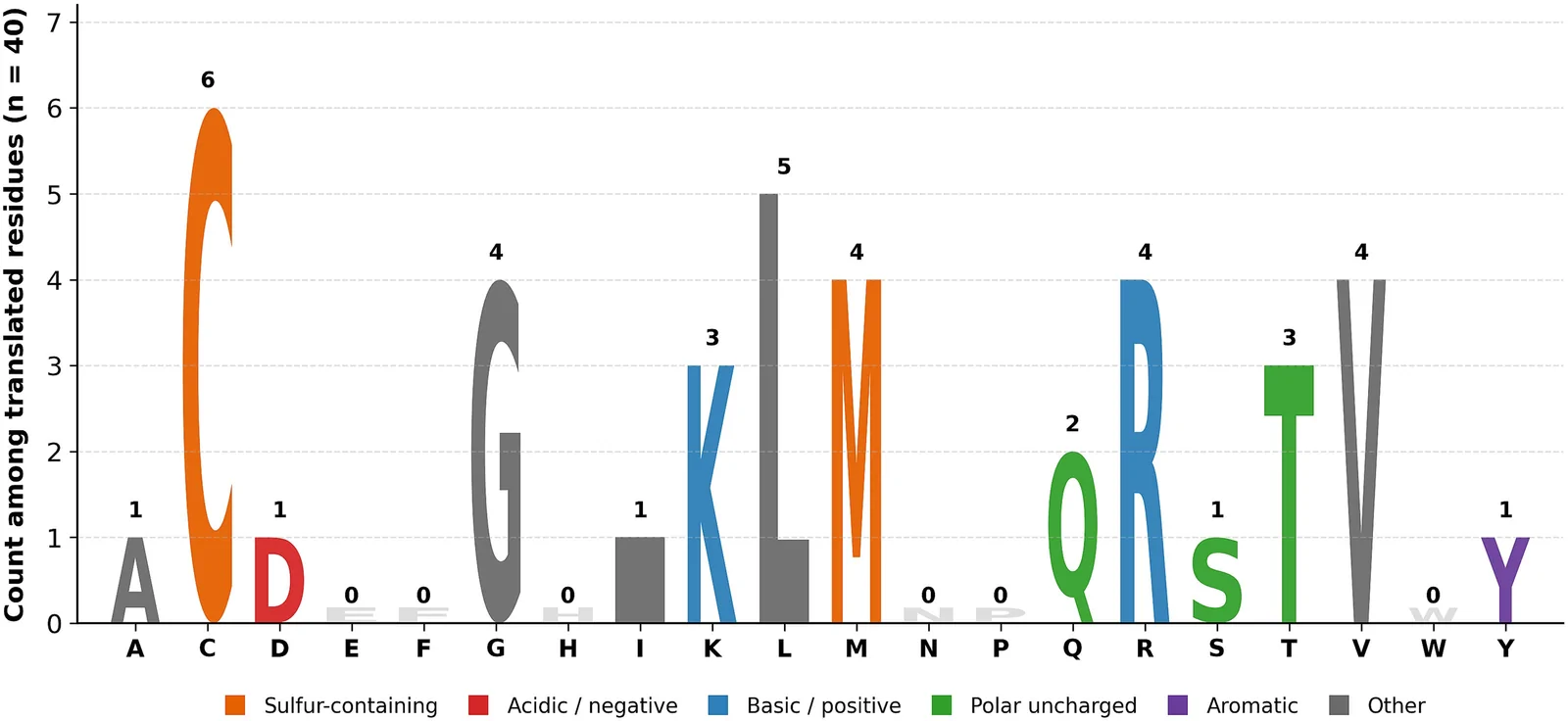
Amino-acid composition of translated top 20 6-mer; letter height represents the count of each amino acid among 40 translated residues; Cys and Met are highlighted in orange, showing significant enrichment of sulfur-containing residues in the top-ranked CWM tokens.

#### Interpretation of position weight function

In DDTRN, **P** represents the learnable parameter tensor associated with the PWFs, which encodes position-dependent information in sequence representation. To investigate how PWF contributes under different rank settings, we analyzed the learned |*P_pqh_*| values on the Dream5_Ecoli dataset from rank 1 to rank 6, using it to measure the relative contribution strength of each periodic basis term. Different from the main benchmark experiments, the mode was set to “nonlinear” in this analysis. In nonlinear mode, tokens of length rank are extracted without redundant sliding windows, so |*P_pqh_*| reflects the contribution of positional information at the token level rather than at each single-base position. Each coefficient *P_pqh_* ​corresponds to a cosine basis term with a period defined in Equation (2). Therefore, a larger |*P_pqh_*| value indicates that the corresponding periodic component contributes more strongly to sequence encoding. Supplementary File S1 provides statistics for the |*P_pqh_*| values of periodic terms 2-1001 across different ranks.

Figure 6 displays the top 20 periodic terms ranked by |*P_pqh_*| magnitude for ranks 1 through 6. It can be seen that at lower ranks (*rank* = 1, 2, 3), the |*P_pqh_*| values for the top 20 terms are significantly higher than those at higher ranks (*rank* = 4, 5, 6). This suggests that when the local contextual information carried by each low-rank fragment is limited, the model relies more heavily on high-magnitude periodic terms to encode the sequence, consistent with the contribution of PWF observed in the parameter-freezing experiments. As rank increases, the overall magnitude of |*P_pqh_*| tends to stabilize because higher-rank fragments inherently contain richer sequence information, reducing the model’s reliance on specific periodic contributions for enhancement. This finding also supports the conclusion that at higher ranks, the performance gain from introducing positional information via PWF in the DD model is relatively limited, thereby narrowing the advantage over ML methods based solely on *k*-mer frequency features. Additionally, significant differences in |*P_pqh_*| values among the top 20 periodic terms across different ranks are observable, indicating that the model captures distinct sequence structural features at different rank granularities. For completeness of the analysis, Supplementary Fig. S4 and Supplementary File S2 also provide the |*P_pqh_*| value analysis for the linear mode.

**Figure 6:**
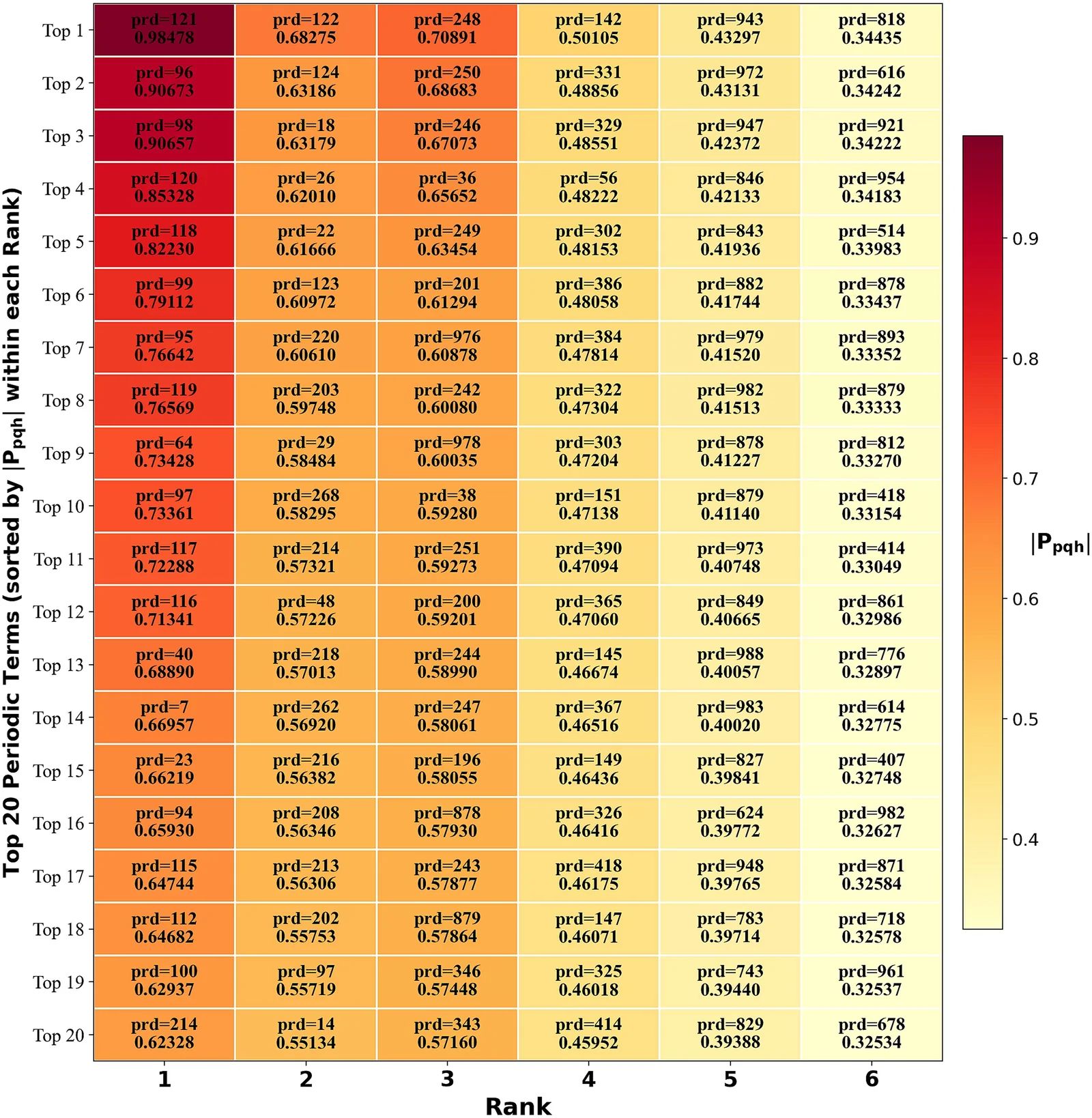
Distribution of |*P_pqh_*| values for periodic terms in the PWF of DD across different rank settings (nonlinear mode); the heatmap shows the top 20 periodic terms sorted by the absolute value of the elements in tensor **P** (namely |*P_pqh_*|) across ranks 1–6; “Prd” indicates the corresponding periodic terms defined in Equation (2); a larger |*P_pqh_*| value indicates greater contribution from the corresponding periodicity in the sequence pattern differentiating regulatory and non-regulatory interaction.

### Case demonstration

To further compare the prediction characteristics of DDTRN with those of conventional machine learning models, we selected the three TF genes with the largest numbers of known regulatory relationships in the RegulonDB_Ecoli dataset, namely *crp*, *nac*, and *lrp*, for case study analysis. For each TF, we first compared the top 100 predicted positive targets by DDTRN, SVM, and GBDT. As shown in Fig. 7A, the limited intersections among the top-ranked predictions of the three methods indicate that they prioritize candidate regulatory relationships differently. In particular, the intersections between DDTRN and the two conventional machine learning methods were generally smaller than the intersection between SVM and GBDT, suggesting that SVM and GBDT exhibit relatively similar ranking preferences in this task. These results indicate that DDTRN prioritizes a complementary set of high-ranking candidate regulatory relationships relative to conventional *k*-mer-based ML models.

**Figure 7:**
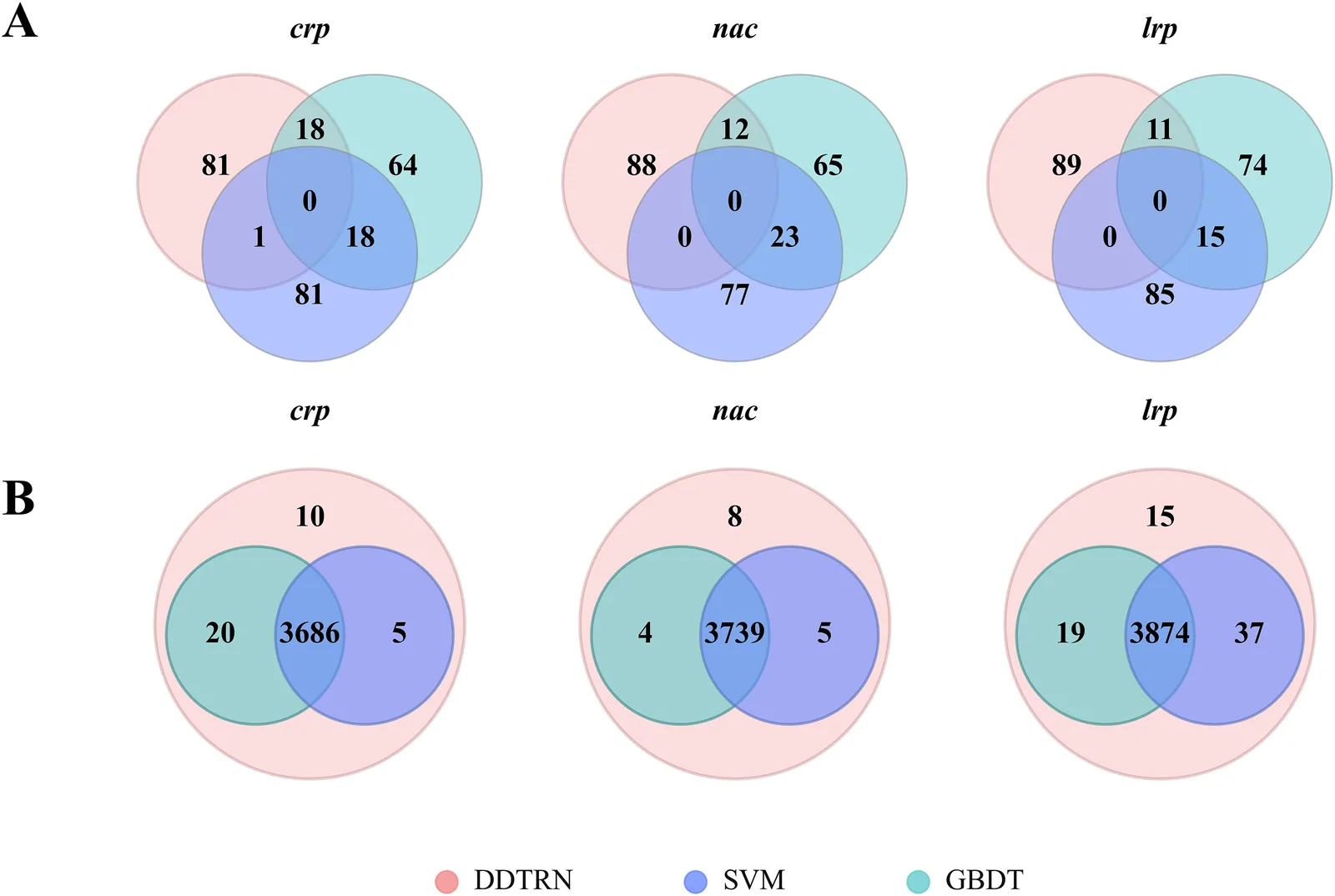
Comparison of top-ranked and overall predicted regulatory targets for TF genes *crp*, *nac*, and *lrp*; Venn diagrams comparing predicted positive targets by DDTRN, SVM, and GBDT for the three TF genes with the largest numbers of known regulatory relationships in the RegulonDB_Ecoli dataset: *crp*, *nac*, and *lrp*; (A) comparison of the top 100 (ranked by model score) predicted targets for each TF gene; (B) comparison of all predicted positive regulatory relationships for each TF; the numbers indicate the counts of candidate TGs in each model-specific or shared region.

When all predicted positive regulatory relationships were considered, a large proportion of candidate interactions were shared by the three methods, indicating overall consistency among their prediction results (Fig. 7B). Meanwhile, DDTRN also identified model-specific candidate regulatory relationships beyond those predicted by SVM and GBDT. These additional predictions may reflect the ability of DDTRN to extract more comprehensive sequence information by integrating both compositional and positional features, thereby capturing regulatory signals that cannot be fully represented by *k*-mer frequency statistics alone.

### Overall prediction

Previous network inference studies have shown that no single inference method performs optimally across all datasets, whereas integrating predictions from multiple methods can improve robustness across diverse datasets [25]. Inspired by this, we retained TF–TG pairs predicted as positive by DDTRN, SVM, and GBDT as higher-confidence candidate regulatory relationships. For *E. coli*, RegulonDB_Ecoli instead of Dream5_Ecoli was selected as the representative dataset, because it contains a larger number of known regulatory relationships, making it more suitable for candidate regulatory relationship mining and consensus screening.

As shown in Table 3, the number of potential regulatory relationships predicted by each model varied across datasets, reflecting differences in feature representation and classification strategies among DDTRN, SVM, and GBDT. To obtain more reliable candidate interactions, we focused on the “All3 Consensus” results, namely TF–TG pairs simultaneously predicted as positive by all three models. The consensus predictions ranged from 4279 pairs in *C. glutamicum* to 89 411 pairs in *B. subtilis*, with 87 624 consensus pairs identified in RegulonDB_Ecoli and 84 420 in *M. tuberculosis*. These jointly predicted relationships are less likely to be driven by the bias of a single model and therefore represent higher-confidence candidates for subsequent experimental validation, TRN annotation, and functional analysis. Complete prediction results are provided in Supplementary Files S3–S9.

**Table 3:** The number of regulatory relationships predicted by the three models across seven datasets.

**Dataset**	**DDTRN**	**SVM**	**GBDT**	**All3 consensus**
RegulonDB_Ecoli	133 626	147 050	138 212	87 624
Subtiwiki_Bsubtilis	139 998	165 889	150 501	89 411
*S. enterica*	23 001	21 814	18 784	15 397
*C. glutamicum*	11 295	12 896	8152	4279
*M. tuberculosis*	126 581	121 142	119 462	84 420
*P. aeruginosa*	52 304	50 947	48 931	37 362
*S. coelicolor*	16 896	19 724	17 710	14 261

## Discussion

In this study, we proposed DDTRN, a sequence-driven framework for predicting bacterial TRNs based on the DD model. Existing TRN inference methods have commonly relied on transcriptomic profiles, graph-based representations, or integrated omics features, which differ substantially in their input requirements from this study. For example, GRADIS and GRGNN rely mainly on transcriptomic information or expression-derived graph representations [12, 13], whereas PGBTR integrates gene-expression and genomic information [14]. Although these methods are useful when sufficient expression data are available, transcriptome-based inference can still be affected by data quality, noise, batch effects, and condition specificity. Benchmark studies have also shown that the performance of gene regulatory network inference is sensitive to data type and evaluation settings [37]. In contrast, DDTRN provides a complementary strategy based solely on gene sequences, especially suitable for bacterial species with limited expression data.

Across eight bacterial benchmark datasets, DDTRN achieved the best average performance among all compared methods, with an average AUROC of 0.8690, AUPR of 0.8683, and F1 score of 0.8053. Compared with conventional machine learning methods based on *k*-mer frequency features, DDTRN showed more stable overall performance across datasets. This result supports the idea that genomic sequences contain regulatory-relevant information. Previous sequence-based models, such as DeepBind, DeepSEA, DanQ, and Enformer, have demonstrated that DNA sequences can encode information related to binding specificity, regulatory sequence code, motif grammar, and gene expression patterns [20–23]. However, compared with many deep learning models whose internal representations are often difficult to interpret, DDTRN retains a more explicit and interpretable representation through the DD architecture. Specifically, the CWM captures compositional information of sequence tokens, whereas the PWF introduces position-dependent or arrangement-related information, consistent with the theoretical basis of the DD method [30].

The parameter sensitivity analyses showed that DDTRN maintained stable performance under different settings of rank, vec_dim, and num_basis. In particular, DDTRN showed a more obvious advantage at lower ranks, where short *k*-mers contain limited contextual information. This suggests that PWF can compensate for insufficient context information by introducing position-dependent weighting. As rank increases, *k*-mer tokens themselves contain richer sequence patterns, reducing the relative contribution of PWF and narrowing the performance gap between DDTRN and conventional *k*-mer frequency-based methods. The robustness analysis further showed that DDTRN can maintain reliable predictive performance when the amount of training data is reduced, suggesting its potential utility in limited-sample scenarios.

In addition to predictive performance, DDTRN provides interpretable model parameters. The PWF analysis showed that positional periodic components contributed differently across rank settings, with stronger contributions at lower ranks and more stable values at higher ranks. This supports the view that PWF provides additional positional information when the token length is short. The CWM analysis further identified high-norm 6-mer tokens that contributed strongly to sequence representation. Translation-based composition analysis further showed that the top 20 high-norm 6-mers were significantly enriched in sulfur-containing amino-acid-associated codon patterns, suggesting a non-random compositional preference among the strongest CWM tokens. The preference in amino acid composition may be associated with certain structural features of TFs [38], and the underlying molecular mechanisms require further investigation.

We also applied DDTRN together with SVM and GBDT to predict candidate TF–TG relationships across seven datasets and identified consensus regulatory relationships supported by all three models. Because DDTRN, SVM, and GBDT use different sequence representations and classification strategies, their shared positive predictions are more likely to represent cross-model regulatory signals rather than model-specific biases. In addition, curated bacterial regulatory resources such as RegulonDB, RegPrecise, DBTBS, and CoryneRegNet show that bacterial TRN annotation is a continuous process that combines literature curation, experimental evidence, and comparative genomics [39–42]. Therefore, the consensus TF–TG pairs identified in this study may serve as prioritized candidates for future experimental validation and bacterial TRN annotation.

Despite these advantages, several limitations of DDTRN should be acknowledged. First, bacterial transcriptional regulation is influenced by a variety of factors, including TF activity, environmental signals, cofactors, promoter architecture, nucleoid-associated proteins, and combinatorial regulation [43]. Therefore, sequence information alone cannot fully reflect the dynamic regulatory state of a cell. Second, current bacterial TRN annotations remain incomplete even in well-curated resources, and negative samples generated from currently unannotated TF–TG pairs may include undiscovered regulatory interactions. This limitation may constrain supervised learning performance. Future work may further evaluate topology-aware negative sampling strategies, such as degree distribution balanced sampling, to reduce degree-related sampling bias in biological networks [44]. Third, the current DDTRN framework uses concatenated gene sequences rather than experimentally defined promoter regions or TF-binding regions. Future work should incorporate more precise regulatory regions, such as promoter sequences, upstream regions around transcription start sites, or experimentally supported TFBSs. In the future, DDTRN can be extended from a sequence-driven framework to a multimodal regulatory prediction framework, integrating multi-omics and transcriptional regulatory data, which is expected to further improve the accuracy and biological relevance of bacterial TRN reconstruction.

Overall, DDTRN provides a scalable, interpretable, and data-efficient sequence-based framework for bacterial TRN prediction. It complements existing expression-based and graph-based approaches by enabling regulatory relationship prediction in species where large-scale omics data (such as transcriptome and proteome) are limited. With further integration of experimentally defined regulatory regions and multimodal regulatory evidence, DDTRN has the potential to become a more powerful tool for bacterial TRN annotation, candidate regulatory interaction discovery, and functional genomics studies.

**Key Points**

- DDTRN introduces a Dual Descriptor framework that jointly encodes sequence composition and positional arrangement from concatenated transcription factor–target gene pairs, overcoming the limitation of pure *k*-mer frequency features.
- On eight benchmark bacterial datasets, DDTRN achieves average area under the receiver operating characteristic curve of 0.869 and area under the precision–recall curve of 0.868, outperforming six conventional machine learning methods, with particularly strong gains at low *k*-mer ranks.
- The model demonstrates robust performance across a wide range of hyperparameters and with as little as 20%–30% of training data, making it well suited for non-model organisms with limited transcriptomic data.
- DDTRN offers interpretable parameters that reveal biologically meaningful sequence motifs and positional periodicities, and its consensus predictions with support vector machine/Gradient Boosting Decision Trees provide high-confidence candidates for experimental validation.

## Supplementary Material

Supplementary_Data_bbag534

Supplementary_File_S1_bbag534

Supplementary_File_S2_bbag534

Supplementary_File_S3_Ecoli_bbag534

Supplementary_File_S4_Bsubt_bbag534

Supplementary_File_S5_Sente_bbag534

Supplementary_File_S6_Cglut_bbag534

Supplementary_File_S7_Mtube_bbag534

Supplementary_File_S8_Paeru_bbag534

Supplementary_File_S9_Scoel_bbag534

## Data Availability

The source code and datasets used in this study are available on GitHub (https://github.com/mbglab/DualDescriptor/tree/main/Apps/DDTRN).
