# Supplementary material for: DDTRN: predicting bacterial transcriptional regulatory networks based on gene sequences using dual descriptor
Source: Brief Bioinform. 2026 Sep 29;27(5):bbag534. doi: 10.1093/bib/bbag534 (PMC13619341; doi:10.1093/bib/bbag534)
Supplement: Supplementary file 1 [file supplementary_data_bbag534.pdf]

Supplementary Data

DDTRN: Predicting Bacterial Transcriptional Regulatory Networks Based on Gene Sequences using Dual Descriptor

Pu Nie, Bin-Guang Ma*

Hubei Key Laboratory of Agricultural Bioinformatics, College of Informatics, Huazhong Agricultural University, Wuhan 430070, China

*To whom correspondence should be addressed. Tel & Fax: +86 2787280877.

E-mail address: mbg@mail.hzau.edu.cn (Bin-Guang Ma)

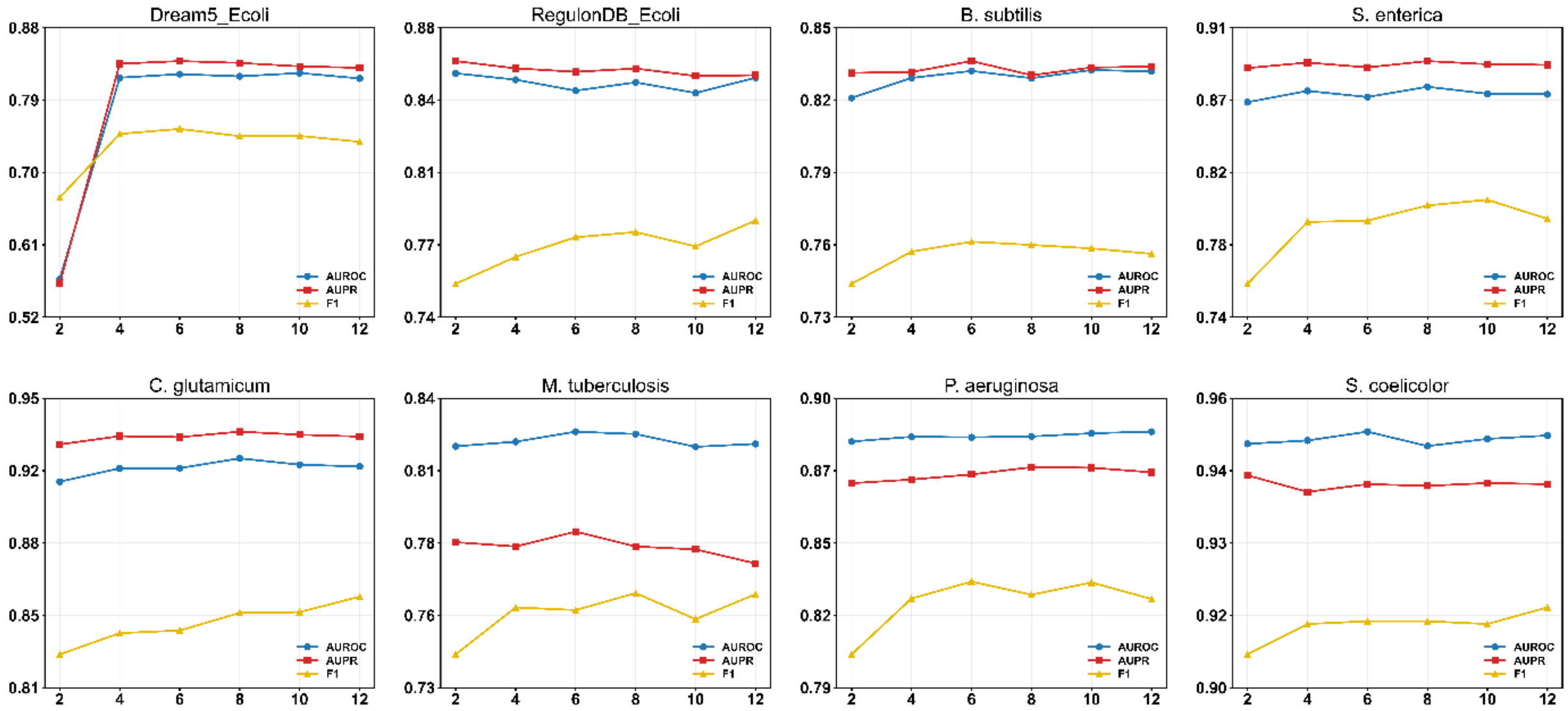

Figure S1. Sensitivity analysis of the vec_dim parameter. Performance generally increases with vec_dim and *vec_dim* = 10 is adopted to show the prediction performance.

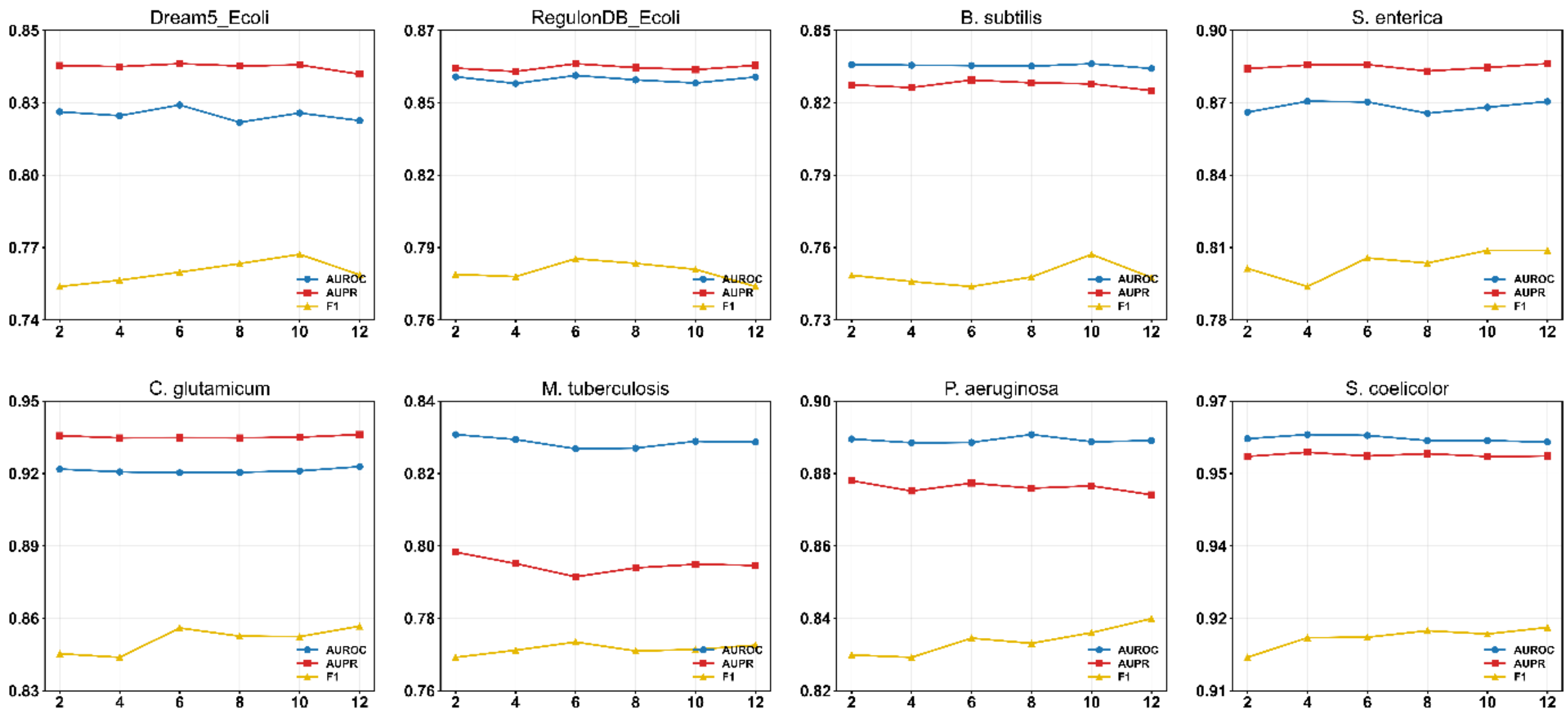

Figure S2. Sensitivity analysis of the num_basis parameter. Generally, DDTRN is not quite sensitive to num_basis variation and *num_basis* = 10 is adopted to show the prediction performance.

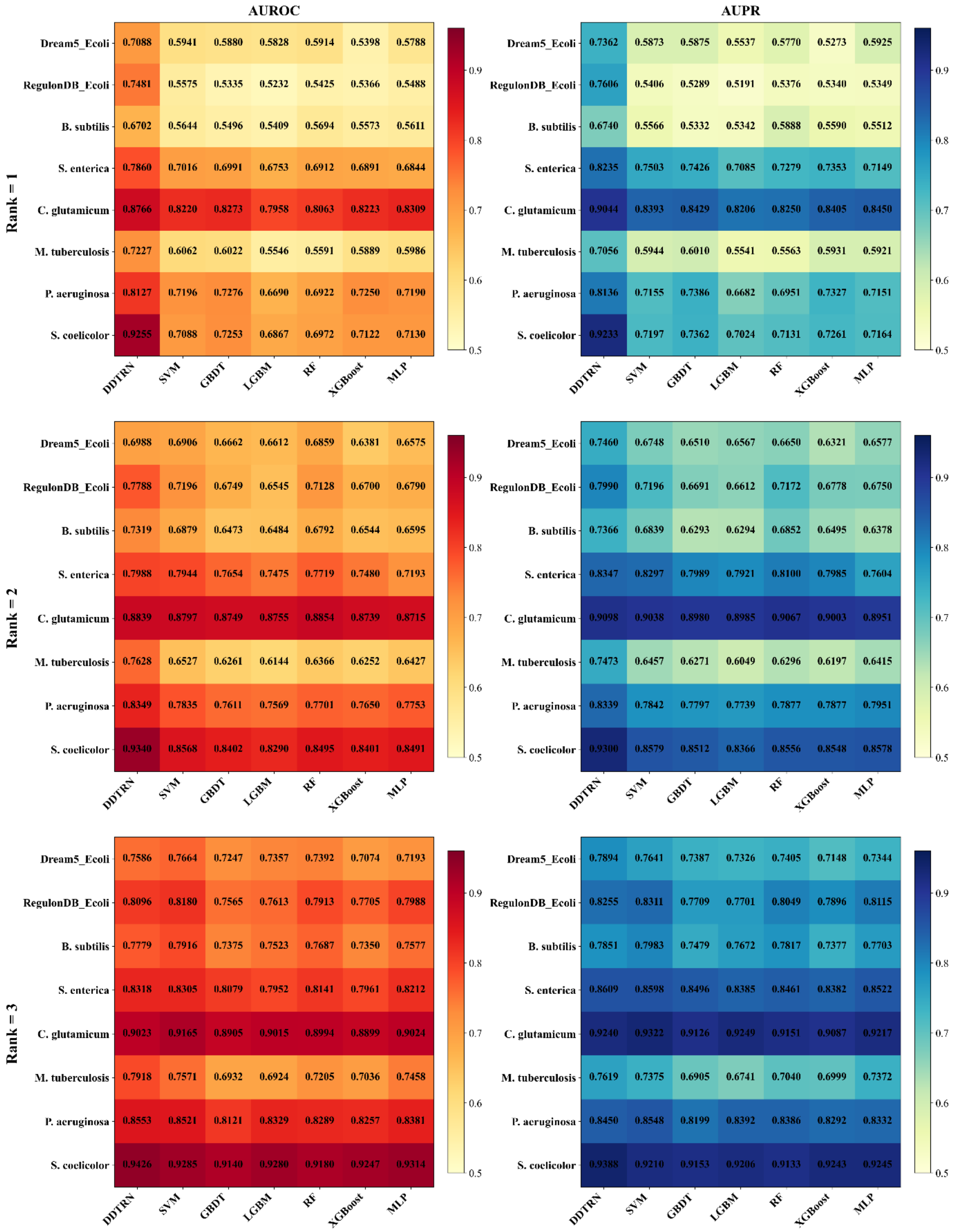
AUROC
AUPR
Rank = 1
Rank = 2
Rank = 3
DDTRN SVM GBDT LGBM RF XGBoost MLP
Dream5_Ecoli 0.7088 0.5941 0.5880 0.5828 0.5914 0.5398 0.5788
RegulonDB_Ecoli 0.7481 0.5575 0.5335 0.5232 0.5425 0.5366 0.5488
B. subtilis 0.6702 0.5644 0.5496 0.5409 0.5694 0.5573 0.5611
S. enterica 0.7860 0.7016 0.6991 0.6753 0.6912 0.6891 0.6844
C. glutamicum 0.8766 0.8220 0.8273 0.7958 0.8063 0.8223 0.8309
M. tuberculosis 0.7227 0.6062 0.6022 0.5546 0.5591 0.5889 0.5986
P. aeruginosa 0.8127 0.7196 0.7276 0.6690 0.6922 0.7250 0.7190
S. coelicolor 0.9255 0.7088 0.7253 0.6867 0.6972 0.7122 0.7130
Dream5_Ecoli 0.7362 0.5873 0.5875 0.5537 0.5770 0.5273 0.5925
RegulonDB_Ecoli 0.7606 0.5406 0.5289 0.5191 0.5376 0.5340 0.5349
B. subtilis 0.6740 0.5566 0.5332 0.5342 0.5888 0.5590 0.5512
S. enterica 0.8235 0.7503 0.7426 0.7085 0.7279 0.7353 0.7149
C. glutamicum 0.9044 0.8393 0.8429 0.8206 0.8250 0.8405 0.8450
M. tuberculosis 0.7056 0.5944 0.6010 0.5541 0.5563 0.5931 0.5921
P. aeruginosa 0.8136 0.7155 0.7386 0.6682 0.6951 0.7327 0.7151
S. coelicolor 0.9233 0.7197 0.7362 0.7024 0.7131 0.7261 0.7164
Dream5_Ecoli 0.6988 0.6906 0.6662 0.6612 0.6859 0.6381 0.6575
RegulonDB_Ecoli 0.7788 0.7196 0.6749 0.6545 0.7128 0.6700 0.6790
B. subtilis 0.7319 0.6879 0.6473 0.6484 0.6792 0.6544 0.6595
S. enterica 0.7988 0.7944 0.7654 0.7475 0.7719 0.7480 0.7193
C. glutamicum 0.8839 0.8797 0.8749 0.8755 0.8854 0.8739 0.8715
M. tuberculosis 0.7628 0.6527 0.6261 0.6144 0.6366 0.6252 0.6427
P. aeruginosa 0.8349 0.7835 0.7611 0.7569 0.7701 0.7650 0.7753
S. coelicolor 0.9340 0.8568 0.8402 0.8290 0.8495 0.8401 0.8491
Dream5_Ecoli 0.7460 0.6748 0.6510 0.6567 0.6650 0.6321 0.6577
RegulonDB_Ecoli 0.7990 0.7196 0.6691 0.6612 0.7172 0.6778 0.6750
B. subtilis 0.7366 0.6839 0.6293 0.6294 0.6852 0.6495 0.6378
S. enterica 0.8347 0.8297 0.7989 0.7921 0.8100 0.7985 0.7604
C. glutamicum 0.9098 0.9038 0.8980 0.8985 0.9067 0.9003 0.8951
M. tuberculosis 0.7473 0.6457 0.6271 0.6049 0.6296 0.6197 0.6415
P. aeruginosa 0.8339 0.7842 0.7797 0.7739 0.7877 0.7877 0.7951
S. coelicolor 0.9300 0.8579 0.8512 0.8366 0.8556 0.8548 0.8578
Dream5_Ecoli 0.7586 0.7664 0.7247 0.7357 0.7392 0.7074 0.7193
RegulonDB_Ecoli 0.8096 0.8180 0.7565 0.7613 0.7913 0.7705 0.7988
B. subtilis 0.7779 0.7916 0.7375 0.7523 0.7687 0.7350 0.7577
S. enterica 0.8318 0.8305 0.8079 0.7952 0.8141 0.7961 0.8212
C. glutamicum 0.9023 0.9165 0.8905 0.9015 0.8994 0.8899 0.9024
M. tuberculosis 0.7918 0.7571 0.6932 0.6924 0.7205 0.7036 0.7458
P. aeruginosa 0.8553 0.8521 0.8121 0.8329 0.8289 0.8257 0.8381
S. coelicolor 0.9426 0.9285 0.9140 0.9280 0.9180 0.9247 0.9314
Dream5_Ecoli 0.7894 0.7641 0.7387 0.7326 0.7405 0.7148 0.7344
RegulonDB_Ecoli 0.8255 0.8311 0.7709 0.7701 0.8049 0.7896 0.8115
B. subtilis 0.7851 0.7983 0.7479 0.7672 0.7817 0.7377 0.7703
S. enterica 0.8609 0.8598 0.8496 0.8385 0.8461 0.8382 0.8522
C. glutamicum 0.9240 0.9322 0.9126 0.9249 0.9151 0.9087 0.9217
M. tuberculosis 0.7619 0.7375 0.6905 0.6741 0.7040 0.6999 0.7372
P. aeruginosa 0.8450 0.8548 0.8199 0.8392 0.8386 0.8292 0.8332
S. coelicolor 0.9388 0.9210 0.9153 0.9206 0.9133 0.9243 0.9245
0.9 0.8 0.7 0.6 0.5

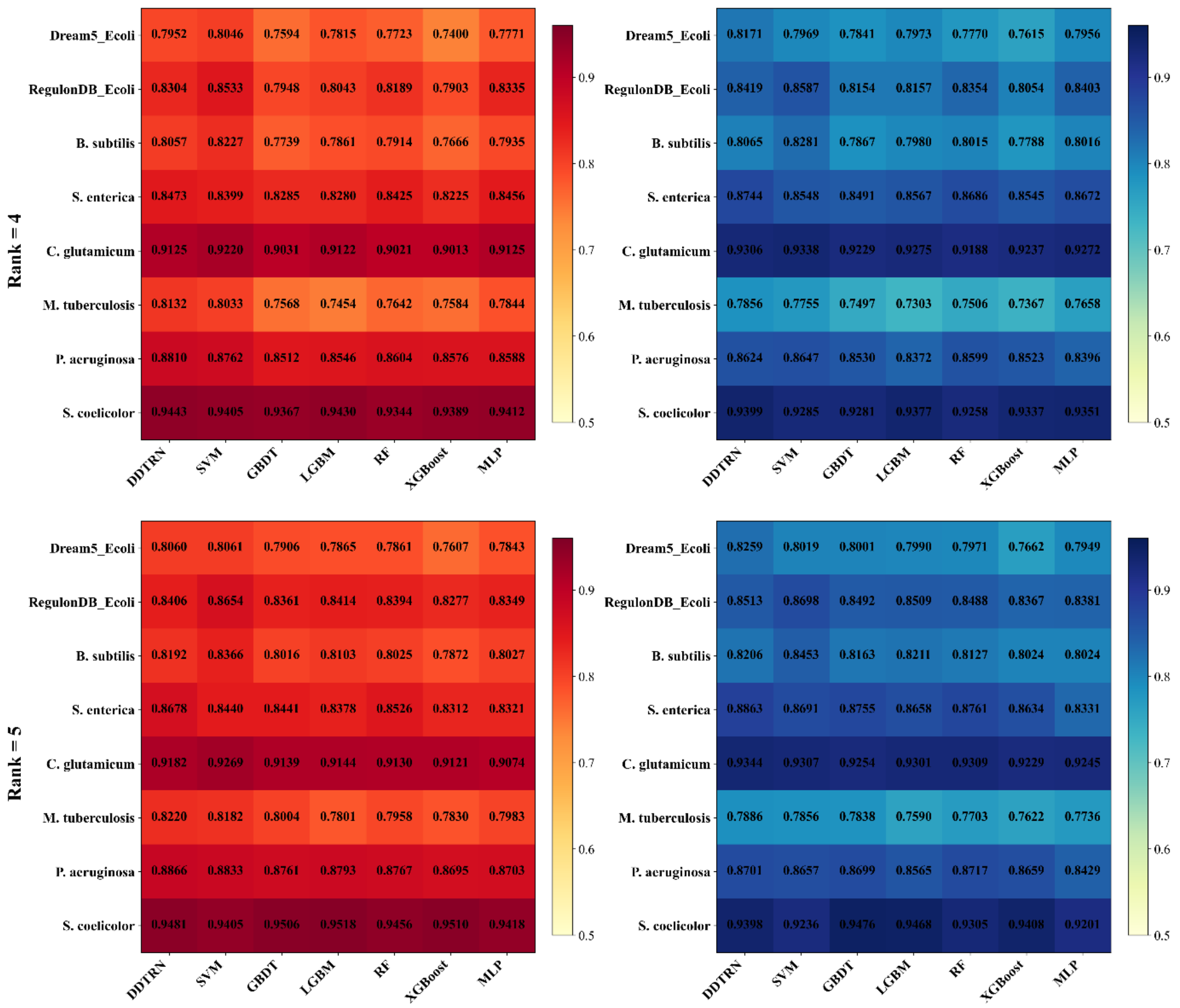

Figure S3. Heatmaps showing the AUROC and AUPR scores of different methods on the benchmark datasets at ranks 1 to 5. AUROC and AUPR are shown side by side for all methods across eight benchmark datasets. Color intensity increases with higher metric value.

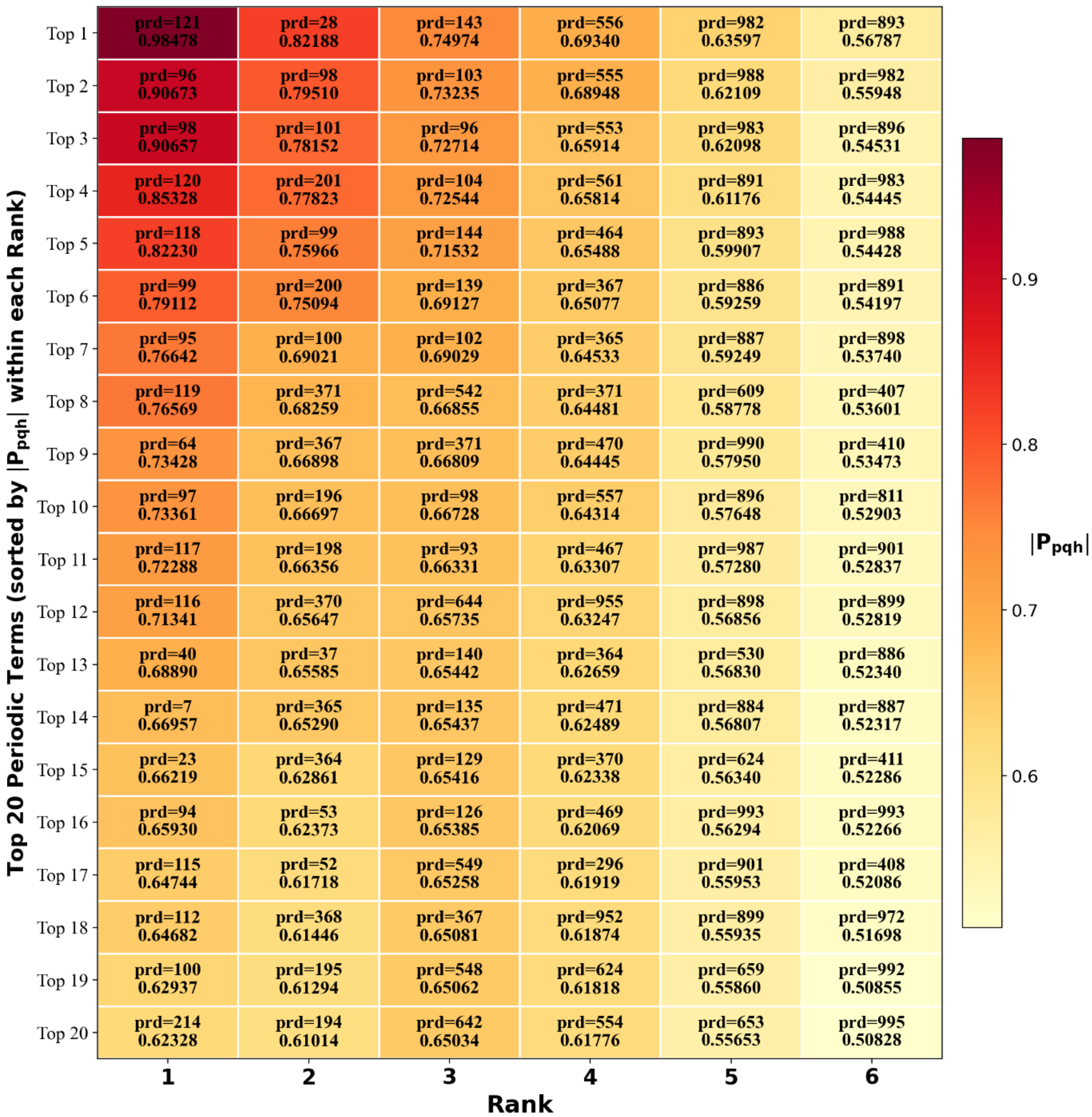

Figure S4. Distribution of |P_{pgh}| values for periodic terms in the PWF of DDTRN across different rank settings (Linear mode). Heatmap shows the top 20 periodic terms sorted by the absolute value of the elements in tensor **P** (namely |P_{pqh}|) across ranks 1 to 6. "prd" indicates the corresponding periodic terms defined in Eq. (2). Larger |P_{pqh}| value indicates greater contribution from the corresponding periodicity in the sequence pattern differentiating Regulatory and Non-regulatory interaction.

Table S1. Performance of DDTRN and baseline methods over 20 repeated runs

Dataset	Metric	DDTRN	SVM	GBDT	LGBM	RF	XGBoost	MLP
Dream5_Ecoli	AUROC	**0.8053 ± 0.0172**	0.7836 ± 0.0166	0.7765 ± 0.0192	0.7763 ± 0.0180	0.7760 ± 0.0168	0.7620 ± 0.0158	0.7548 ± 0.0167
	AUPR	**0.8162 ± 0.0168**	0.7945 ± 0.0212	0.7961 ± 0.0208	0.7897 ± 0.0230	0.7978 ± 0.0190	0.7842 ± 0.0167	0.7657 ± 0.0214
	F1	**0.7253 ± 0.0236**	0.7200 ± 0.0198	0.6921 ± 0.0203	0.7029 ± 0.0196	0.6947 ± 0.0189	0.6864 ± 0.0199	0.6887 ± 0.0157
RegulonDB_Ecoli	AUROC	0.8514 ± 0.0082	**0.8665 ± 0.0078**	0.8483 ± 0.0093	0.8474 ± 0.0093	0.8500 ± 0.0093	0.8381 ± 0.0090	0.8438 ± 0.0067
	AUPR	0.8527 ± 0.0121	**0.8713 ± 0.0095**	0.8578 ± 0.0115	0.8531 ± 0.0100	0.8560 ± 0.0115	0.8473 ± 0.0104	0.8456 ± 0.0101
	F1	0.7797 ± 0.0114	**0.7951 ± 0.0092**	0.7652 ± 0.0120	0.7710 ± 0.0093	0.7620 ± 0.0107	0.7611 ± 0.0105	0.7711 ± 0.0087
Subtiwiki_Bsubtilis	AUROC	0.8197 ± 0.0122	**0.8238 ± 0.0121**	0.8088 ± 0.0118	0.8104 ± 0.0121	0.8132 ± 0.0120	0.7899 ± 0.0125	0.7841 ± 0.0104
	AUPR	0.8182 ± 0.0158	**0.8305 ± 0.0133**	0.8195 ± 0.0140	0.8208 ± 0.0129	0.8251 ± 0.0119	0.8086 ± 0.0132	0.7828 ± 0.0154
	F1	0.7439 ± 0.0115	**0.7538 ± 0.0121**	0.7265 ± 0.0168	0.7360 ± 0.0129	0.7197 ± 0.0165	0.7136 ± 0.0162	0.7203 ± 0.0099
S. enterica	AUROC	**0.8801 ± 0.0129**	0.8670 ± 0.0130	0.8699 ± 0.0121	0.8558 ± 0.0136	0.8701 ± 0.0124	0.8527 ± 0.0142	0.8363 ± 0.0146
	AUPR	**0.8906 ± 0.0169**	0.8707 ± 0.0204	0.8819 ± 0.0151	0.8648 ± 0.0193	0.8818 ± 0.0141	0.8687 ± 0.0159	0.8312 ± 0.0282
	F1	**0.8255 ± 0.0132**	0.8129 ± 0.0102	0.7938 ± 0.0105	0.7864 ± 0.0107	0.7830 ± 0.0122	0.7797 ± 0.0135	0.7737 ± 0.0127
C. glutamicum	AUROC	0.9129 ± 0.0077	**0.9147 ± 0.0072**	0.9143 ± 0.0090	0.9131 ± 0.0072	0.9118 ± 0.0072	0.9085 ± 0.0082	0.9023 ± 0.0075
	AUPR	**0.9308 ± 0.0065**	0.9293 ± 0.0082	0.9297 ± 0.0069	0.9285 ± 0.0079	0.9273 ± 0.0055	0.9264 ± 0.0070	0.9199 ± 0.0078
	F1	0.8364 ± 0.0102	**0.8465 ± 0.0094**	0.8401 ± 0.0120	0.8410 ± 0.0122	0.8332 ± 0.0099	0.8363 ± 0.0096	0.8277 ± 0.0071
M. tuberculosis	AUROC	**0.8260 ± 0.0063**	0.8240 ± 0.0052	0.8126 ± 0.0056	0.7902 ± 0.0072	0.8072 ± 0.0050	0.8002 ± 0.0062	0.7952 ± 0.0089
	AUPR	**0.7875 ± 0.0088**	0.7873 ± 0.0071	0.7865 ± 0.0069	0.7622 ± 0.0082	0.7774 ± 0.0069	0.7737 ± 0.0067	0.7667 ± 0.0108
	F1	0.7653 ± 0.0078	**0.7714 ± 0.0045**	0.7413 ± 0.0064	0.7262 ± 0.0061	0.7372 ± 0.0067	0.7357 ± 0.0063	0.7327 ± 0.0088
P. aeruginosa	AUROC	**0.8964 ± 0.0081**	0.8922 ± 0.0062	0.8945 ± 0.0061	0.8950 ± 0.0063	0.8959 ± 0.0062	0.8899 ± 0.0072	0.8748 ± 0.0089
	AUPR	0.8836 ± 0.0102	0.8737 ± 0.0090	0.8851 ± 0.0077	0.8811 ± 0.0093	**0.8862 ± 0.0091**	0.8810 ± 0.0100	0.8477 ± 0.0145
	F1	0.8258 ± 0.0090	**0.8309 ± 0.0076**	0.8225 ± 0.0089	0.8299 ± 0.0093	0.8187 ± 0.0103	0.8223 ± 0.0101	0.8160 ± 0.0095
S. coelicolor	AUROC	0.9551 ± 0.0043	0.9469 ± 0.0057	**0.9566 ± 0.0045**	0.9547 ± 0.0053	0.9536 ± 0.0052	0.9547 ± 0.0047	0.9460 ± 0.0077
	AUPR	0.9437 ± 0.0052	0.9351 ± 0.0073	**0.9461 ± 0.0080**	0.9436 ± 0.0086	0.9411 ± 0.0086	0.9429 ± 0.0077	0.9276 ± 0.0134
	F1	0.9161 ± 0.0057	0.9014 ± 0.0058	**0.9169 ± 0.0053**	0.9164 ± 0.0062	0.9130 ± 0.0057	0.9143 ± 0.0048	0.9042 ± 0.0073
**Average**	AUROC	**0.8684**	0.8649	0.8602	0.8554	0.8597	0.8495	0.8422
	AUPR	**0.8654**	0.8616	0.8628	0.8555	0.8616	0.8541	0.8359
	F1	0.8023	**0.8040**	0.7873	0.7887	0.7827	0.7812	0.7793

Note: Random seeds 0-19 were used for all the methods in these repeated runs.

Table S2. Parameter-freezing experiments of DDTRN

Rank	Metric	DDTRN	fixed CWM	fixed PWF
	AUROC	**0.7088**	0.6069	0.5880
1	AUPR	**0.7362**	0.6276	0.6008
	F1	0.6412	**0.6661**	**0.6661**
	AUROC	**0.6988**	0.5784	0.5516
2	AUPR	**0.7460**	0.6088	0.5824
	F1	0.6286	**0.6661**	**0.6661**
	AUROC	**0.7586**	0.6711	0.5194
3	AUPR	**0.7894**	0.7062	0.5372
	F1	**0.6960**	0.6366	0.6661
	AUROC	**0.7952**	0.6945	0.4959
4	AUPR	**0.8171**	0.7171	0.5109
	F1	**0.7152**	0.6350	0.6661
	AUROC	**0.8060**	0.5235	0.5191
5	AUPR	**0.8259**	0.5358	0.5311
	F1	**0.7181**	0.6661	0.6661
	AUROC	**0.8213**	0.7146	0.8136
6	AUPR	0.8296	0.7294	**0.8312**
	F1	**0.7429**	0.6690	0.7059

Note: Parameter-freezing experiments were conducted on the Dream5_Ecoli dataset.
